# Pore-Scale Study of the Effect of Through-Plane Carbon Support Gradients on Catalyst Layer Performance in Proton Exchange Membrane Fuel Cells

**DOI:** 10.3390/membranes16100326

**Published:** 2026-09-29

**Authors:** Siyu Yang, Hao Wang

**Affiliations:** 1 Naval Architecture and Ocean Engineering College, Dalian Maritime University, Dalian 116026, China; jackyang_dlmu@163.com; 2 College of Transportation Engineering, Dalian Maritime University, Dalian 116026, China

**Keywords:** Lattice Boltzmann method, proton exchange membrane fuel cell, catalyst layer, carbon distribution gradient, oxygen transport, electrochemical performance

## Abstract

The cathode catalyst layer (CL) is a key component governing the performance of proton exchange membrane fuel cells (PEMFCs), yet the trade-off between oxygen transport and electrochemical activity in conventional uniform CLs remains a critical limitation. This study presents a pore-scale numerical investigation into the effect of through-plane carbon support volume fraction gradients on oxygen transport and electrochemical performance in PEMFC catalyst layers. A lattice Boltzmann method (LBM) model, coupled with a stochastic reconstruction algorithm, was employed to simulate three gradient configurations, linear (LG), stepwise (SG), and hybrid stepwise (HSG), under varying gradient directions and magnitudes. The results demonstrate that positive gradients, with higher carbon fraction near the membrane, enhance oxygen diffusivity and electrochemical activity compared to negative and uniform configurations. Among all designs, the HSG with a positive large gradient achieves the highest peak current density of 694.11 mA cm^−2^, representing a 13.4% increase over the uniform structure. This improvement is attributed to the optimized oxygen concentration distribution and electrochemically active surface area, enabled by a broadened pore size distribution that facilitates gas transport while concentrating catalytic activity in the membrane-adjacent region. The findings establish a quantitative link between carbon support gradient design and CL performance, providing a simulation-based framework for the rational engineering of high-performance PEMFC electrodes.

## 1. Introduction

Amid the accelerating global transition toward sustainable energy systems, technological innovation in clean and renewable energy has become a fundamental enabler of decarbonization and long-term environmental resilience [1]. Within this paradigm, proton exchange membrane fuel cells (PEMFCs) are increasingly recognized as a transformative energy conversion technology capable of displacing conventional fossil fuel systems. Distinguished by their zero carbon emissions, low acoustic footprint, and energy conversion efficiencies exceeding 60%, PEMFCs offer an attractive pathway for integrating hydrogen-based energy solutions into next-generation power infrastructures [2]. These electrochemical devices convert chemical energy directly into electricity via hydrogen oxidation, producing only water as a benign byproduct, thus aligning closely with global carbon neutrality objectives. The high performance and modularity of PEMFCs have stimulated wide interest in applications spanning green transportation, distributed renewable power systems, and large-scale energy storage [3,4,5]. However, their commercial scalability is hindered by persistent challenges in cathode catalyst layer (CCL) performance, particularly related to mass transport resistance and suboptimal electrochemical activity. A growing body of evidence indicates that the structural morphology of the catalyst layer governs reactant accessibility, ionic and electronic conductivity, and the development of effective triple-phase boundaries [6,7,8]. Conventional homogeneous catalyst layers often exhibit uniform porosity and catalyst distribution, which compromise reactant diffusion and catalyst utilization under high current densities [9,10]. In response, recent advances in graded catalyst layer design have focused on engineering through-plane heterogeneities in porosity, ionomer content, and catalyst dispersion to optimize mass transport and local reaction kinetics. Such architected structures offer a compelling approach to unlocking the full potential of PEMFCs as a high-efficiency platform for renewable energy conversion and utilization [11,12].

Experimental investigations have demonstrated that gradient CLs can substantially enhance oxygen reduction reaction (ORR) kinetics and liquid water removal efficacy through hierarchical manipulation of platinum (Pt) loading or ionomer content within the layered structure [13,14]. To address nonuniform current density distributions and suboptimal catalyst utilization, Zhang et al. systematically explored gradient-distributed cathode catalyst loadings aligned with gas flow channels in PEMFCs. Their findings revealed that an optimized gradient design, characterized by progressively increasing catalyst loading from the channel inlet to outlet, markedly suppresses spatial heterogeneities in current density, thereby improving overall reaction uniformity [15]. Lyu and co-workers further advanced this paradigm by experimentally and computationally investigating hybrid ionomer configurations in gradient CLs. Their work highlighted that integrating fluorinated and non-fluorinated ionomers in a gradient arrangement yields enhanced stability and electrochemical activity under low-humidity conditions, offering innovative material and architectural strategies for durable fuel cell design [16]. Shahgaldi et al. fabricated CLs with dual gradients, namely decreasing ionomer content toward the gas diffusion layer (GDL) and increasing Pt concentration, and assessed their impact on PEMFC performance. The study confirmed that such gradient-engineered CLs expand porosity and enlarge average pore sizes, synergistically optimizing proton conductivity and mass transport. This reciprocal enhancement mitigates ionic resistance while boosting Pt utilization efficiency [17]. Ayoub et al. introduced a scalable two-step coating protocol to fabricate continuously graded ionomer distributions in CLs, evaluating their performance across a broad relative humidity (RH) spectrum. The results underscored the superiority of gradient architectures over homogeneous counterparts, particularly under variable humidity conditions, achieved via precise control of layer-by-layer deposition techniques [18]. Despite these advancements, experimental approaches remain constrained by spatial resolution limitations and challenges in replicating real-operating conditions, which hinder meticulous dissection of how gradient structural parameters modulate multiphysics coupling mechanisms. This underscores the critical need for complementary pore-scale simulations to decipher the intricate interplay between morphology, transport phenomena, and electrochemical performance in gradient CLs.

The inherent limitations of experimental approaches have motivated researchers to employ multi-scale modeling frameworks for elucidating optimization principles of gradient CLs across mesoscopic and microscopic scales, thereby offering theoretical insights to guide experimental design [19,20,21,22]. For instance, Chen et al. adopted a dual-cathode-layer gradient architecture to systematically investigate the interplay between Pt/C ratio and Nafion content gradients in PEMFC performance under low-humidity/high-current-density conditions. Their findings highlighted that preferential enrichment of Nafion content and Pt/C ratio in the membrane-adjacent sub-layer substantially enhances both catalyst activity and mass transport efficiency [23]. In parallel, Wang et al. introduced a triple-gradient synergistic CCL design, demonstrating through numerical simulations that this architecture achieves ~6.8% higher peak power density compared to conventional uniform or in-plane ionomer-graded CLs with identical ionomer loadings. This improvement arises from synergistic enhancements in oxygen diffusivity and proton conductivity within the porous electrode [24].

Addressing catalyst durability challenges, Yu et al. combined mathematical modeling with experimental validation to assess gradient Pt particle sizing strategies in low-Pt-loaded PEMFCs. Their results confirmed that strategic placement of larger Pt particles in high-stress regions effectively suppresses Pt dissolution kinetics, thereby improving catalyst stability [25]. Yang et al. explored the impact of spatial heterogeneities in Pt and ionomer distributions on cold-start capabilities, revealing that gradient architectures optimize ice formation dynamics. Notably, dual-layer and tri-layer gradient designs extended operational robustness at 80 °C while broadening the feasible sub-zero startup regime, offering a viable pathway for low-temperature applications [26]. Venkatesh K et al. investigated the effects of catalyst gradients along the CL thickness and along the channels and their combinations on fuel cell performance and catalyst utilization, and showed that when the total cathode catalyst loading was 0.35 mg/cm^2^, the constraints due to oxygen and ion diffusion were reduced, and a higher loading near the membrane indicated an increase in fuel cell performance and catalyst utilization [27]. Firat et al. developed an improved aggregate model for the study of PEMFC with functionally gradient components, providing results for different catalyst and ionomer loads along the thickness and in-plane directions, and found that higher catalyst and ionomer loads at the membrane/CL interface can improve performance, especially in the ohmic loss range [28].

To overcome the resolution limitations of macroscale continuum models, mesoscopic simulation approaches have become indispensable for directly reconstructing the porous microstructure of PEMFC CLs. Among such methods, the LBM has emerged as a powerful tool for CL microstructure optimization due to its distinctive capabilities in handling complex boundary conditions and multiphase transport phenomena [29,30,31,32,33]. Hou et al. employed an LBM-based pore-scale model to systematically investigate compositional and structural effects in PEMFC CLs. Their study revealed that elevating the platinum-to-carbon (Pt/C) ratio substantially enhances oxygen transport efficiency, while increasing the ionomer-to-carbon (I/C) ratio optimizes performance by expanding the effective surface area of electrochemically active catalyst sites [34]. Li et al. advanced this work by implementing LBM to analyze gradient distributions of ionomer and platinum within CLs. They demonstrated that ionomer concentration gradients strategically balance proton conductivity and oxygen diffusivity, whereas platinum gradients achieve reaction rate homogenization through spatial relocation of catalyst sites [35]. Molaeimanesh et al. utilized three-dimensional LBM coupled with stochastic reconstruction techniques to elucidate how CL morphology—specifically ellipsoidal stretching characteristics—influences mass transport and current density distribution. Their results identified optimal morphological anisotropy that maximizes interfacial current density at the membrane–CL junction, thereby enhancing overall reaction efficiency [36].

Despite notable progress in pore-scale modeling of CLs, existing LBM investigations have primarily focused on homogeneous or oversimplified hierarchical microstructures. Crucially, the carbon support phase, which dominates the volumetric composition of CLs, has received comparatively limited attention in terms of its spatial distribution and its governing role in mass transport and electrochemical behavior [37]. In particular, systematic studies on through-plane graded carbon particle distributions remain strikingly scarce, leaving a critical gap in the rational design of high-performance fuel cell electrodes for renewable energy systems. To address this knowledge void, we present a stochastic microstructure generation framework tailored to simulate CLs with precisely controlled through-plane gradients in carbon support volume fraction. The study explores multiple gradient distribution modalities and quantifies how gradient intensity influences pore morphology, oxygen diffusivity, and local electrochemical activity. By tracking variations in electrochemically active surface area (ECSA), oxygen concentration profiles, and oxygen consumption rates across the CL, the underlying mechanisms through which carbon gradients enhance electrode functionality are elucidated. This work establishes a rigorous pore-scale methodology for coupling structural design with performance optimization, providing fundamental insights for the targeted engineering of CL architectures. The findings offer a promising route to advance PEMFC technology as a core enabler of efficient and scalable renewable energy conversion.

## 2. Methodology

### 2.1. Microscopic Random Reconstruction Method of Gradient CLs

In this paper, a multi-step method for simulating the random reconstruction of the CL will be adopted. This model integrates the key preparation processes of three components, including the three-dimensional growth simulation of carbon support, the adhesion of Pt particles, and the encapsulation of ionomers. This hierarchical reconstruction strategy establishes a critical link between materials processing and functional performance by accurately capturing: the percolation characteristics of triple-phase boundaries, the tortuosity of oxygen transport pathways, and the ionomer-mediated proton conduction networks. The developed framework represents a significant advancement in pore-scale modeling of PEMFC CLs, offering a quantitative toolset for rational design optimization through structure–property–performance correlations.

The accurate quantification of component volume fractions in CLs necessitates an integrated analysis of critical structural parameters. This study systematically derives volumetric proportions by establishing quantitative relationships among key parameters: Pt loading (*γ_Pt_*), ionomer content (*θ_i_*), Pt-to-carbon mass ratio (*θ_Pt_*_/*C*_), overall CL porosity (*ε_CL_*), and CL thickness (*δ*). Through multi-parameter coupling analysis, the proposed methodology enables precise determination of spatial distribution characteristics for all constituent phases (carbon, platinum particles, and ionomer) while maintaining nanoscale resolution. This framework provides foundational data for rational microstructural design and performance optimization of PEMFC CLs by revealing structure–property–performance interdependencies. The specific calculation method is as follows:

(1) $$\varepsilon_{C}=(1-\varepsilon )\frac{1/\rho_{C}}{\theta_{Pt/C}/\rho_{Pt}+1/\rho_{C}+(1+\theta_{Pt/C})\theta_{i}/(\rho_{i}(1-\theta_{i}))}$$

(2) $$\varepsilon_{Pt}=(1-\varepsilon )\frac{\theta_{Pt/C}/\rho_{C}}{\theta_{Pt/C}/\rho_{Pt}+1/\rho_{C}+(1+\theta_{Pt/C})\theta_{i}/(\rho_{i}(1-\theta_{i}))}$$

(3) $$\varepsilon_{i}=(1-\varepsilon )\frac{\left(1+\theta_{Pt/C})\theta_{i}/(\rho_{i}(1-\theta_{i})\right)}{\theta_{Pt/C}/\rho_{Pt}+1/\rho_{C}+(1+\theta_{Pt/C})\theta_{i}/(\rho_{i}(1-\theta_{i}))}$$

(4) $$\gamma_{Pt}=\varepsilon_{Pt}\rho_{Pt}\delta$$

In the present model, *ε_C_*, *ε_Pt_*, and *ε_i_* denote the volume fractions of the carbon support, Pt particles, and ionomer phases, respectively. The material densities are specified as follows: carbon support (*ρ_C_ =* 1800 mg/cm^3^), Pt particles (*ρ_Pt_ =* 21,450 mg/cm^3^), and ionomer (*ρ_i_ =* 2000 mg/cm^3^). A computational grid resolution of 3 nm is uniformly employed, wherein a single lattice unit (lu) corresponds to a physical dimension of 3 nm, providing a consistent spatial discretization framework for microstructural characterization and parameter quantification. This resolution is consistent with the benchmark studies of Hou et al. [34], in which grid-independence was confirmed by comparing pore-scale results at varying resolutions. The chosen 3 nm resolution sufficiently resolves the key pore size range (20–40 nm in diameter) and the carbon sphere diameter range (8–22 nm), ensuring reliable predictions of pore morphology and transport properties. Basic calculation example simulations were conducted considering standard loading conditions (*γ_Pt_* = 0.3 mg/cm^2^), overall CL porosity (*ε_CL_* = 0.35), Pt-to-carbon mass ratio (*θ_Pt_*_/*C*_ = 0.6), and ionomer content (*θ_i_* = 1:3). Subsequent numerical experiments systematically investigated the influence of carbon support gradient distributions on CL performance metrics, elucidating structure–property relationships governing oxygen transport and electrochemical reaction kinetics within the CL.

#### 2.1.1. Generation of Gradient Carbon Support

Based on the calculations derived from Equations (1)–(4), and utilizing the carbon volume fraction *ε_C_* = 0.3465 reported in the baseline case of Hou et al., three distinct configurations of local carbon volume fraction gradients along the through-plane direction were designed: linear gradient (LG), stepwise gradient (SG), and hybrid stepwise gradient (HSG). These configurations, achieved with carbon sphere diameters ranging from 8 to 22 nm [34], are schematically illustrated in Figure 1a. These configurations establish a hierarchical framework for exploring structure–transport property relationships within the CL, wherein the graded carbon architectures serve as controlled variables in parametric studies.

This study employs a systematic methodology to construct the carbon sphere network model, integrating stochastic generation and iterative optimization. Initially, the number of carbon spheres is estimated based on the target volume fraction of the carbon support. Subsequently, center coordinates and radius parameters for individual spheres are generated using random functions, forming the basis for creating the carbon sphere structure. Corresponding nodes within the computational domain are designated as carbon phase regions. To achieve a gradient distribution of the carbon support volume fraction along the thickness direction, the probability density function governing the spatial distribution of sphere centers is meticulously controlled. The actual volume fraction within the domain is then evaluated to determine its deviation from the target value. If this deviation exceeds ±1%, M is adjusted, and an iterative optimization process is initiated. This process involves continuously generating new parameter combinations until both the volume fraction error and gradient profile requirements are satisfied. Through cyclic execution of this generation, verification, and optimization sequence, the model achieves precise regulation of the target carbon volume fraction, ensuring structural fidelity and accuracy in simulating the CL of PEMFCs.

#### 2.1.2. The Adhesion of Pt Particles and the Coating of Dissociated Ionomers

In the nanoscale CL microstructural reconstruction model developed herein, individual Pt particle dimensions were aligned with the computational lattice resolution and strategically positioned on carbon support surfaces or proximate Pt aggregates. The adhesion dynamics of Pt particles are algorithmically governed as follows: potential adhesion sites, identified as pore coordinates adjacent to existing carbon spheres or Pt particles within the computational domain, are systematically cataloged. A stochastic selection process then converts a fraction of these sites, specifically a subset equivalent to 10% of the total targeted Pt particle count per iteration, into Pt particles. This iterative procedure, cycling through site identification and probabilistic transformation, continues until the requisite Pt loading is achieved. By modulating the spatial dispersion through controlled randomness, this approach faithfully replicates the heterogeneous yet statistically uniform distribution observed in ex situ CL architectures, thereby enhancing the fidelity of pore-scale simulations for PEMFC electrodes.

In this investigation, the spatial distribution of the ionomer phase was modeled using a stochastic probabilistic approach analogous to the Pt particle deposition scheme, aiming to replicate its inherently heterogeneous dispersion. This methodology precludes localized over-aggregation by dynamically adjusting the deposition probability threshold, thereby ensuring a controlled non-uniform distribution. Consequently, it authentically captures the microscale dispersion behavior of ionomers within actual CLs, enhancing the realistic representation of morphological features in PEMFC electrode simulations. It should be noted that the stochastic reconstruction approach adopted in this study is not intended to replicate any specific experimental CL sample, but rather to generate statistically representative microstructures with controlled gradient distributions. The input parameters, including carbon sphere diameters (8–22 nm), Pt/C ratio, and ionomer content, are selected based on typical experimental values reported in the literature [15,17,34]. To ensure structural representativeness, we compare the key morphological metrics of the reconstructed uniform CL, such as pore size distribution (peak at 20–40 nm), overall porosity (~0.35), and ECSA, with those reported in experimental and modeling study [34]. The obtained pore characteristics fall within the typical range of real CLs, and the pore connectivity exceeds 99.6% in all cases, indicating that the reconstructed microstructures preserve the essential transport pathways. For each gradient configuration, a single representative microstructure was generated using a fixed random seed, following the common practice in pore-scale LBM studies of PEMFC CLs [31,33,34,36]. This approach ensures that the observed differences between configurations are primarily attributable to the imposed carbon gradient rather than to random variations in sphere placement. As demonstrated in Table 1, the overall porosity, ECSA, and pore connectivity are remarkably consistent across all configurations (maximum relative deviation in ECSA: 0.41%), confirming that the stochastic reconstruction process does not introduce significant structural discrepancies. The systematic trends observed across multiple gradient types, directions, and magnitudes further support the robustness of our conclusions.

Similar stochastic reconstruction strategies have been widely employed in pore-scale LBM studies of PEMFC CLs [34,36,37], demonstrating good agreement with experimental observations in terms of effective diffusivity and reaction distribution. Therefore, the present gradient structures, while idealized, provide a reliable platform for systematically isolating and quantifying the effects of carbon support gradients on transport and electrochemical performance.

Figure 1b,c depict schematic representations of CL configurations featuring uniform, linear gradient, stepwise gradient, and mixed stepwise gradient. Herein, carbon supports are illustrated in yellow, ionomers in green, and Pt particles in red. These figures comprehensively illustrate, in both 3D and 2D views, the efficacy of the proposed microstructural stochastic reconstruction technique in establishing controlled gradient profiles of carbon volume fraction through the layer thickness. Notably, this method ensures a discrete yet spatially rational arrangement of Pt nanoparticles and ionomer phases within the computational domain, thereby validating its capability to replicate essential structural characteristics of gradient CLs in PEMFC electrodes with high fidelity.

### 2.2. LB Model of Oxygen Transfer and Electrochemical Reactions

LBM bridges the gap between macroscopic continuum theories and microscopic molecular dynamics by integrating statistical mechanics principles, thereby establishing a multiscale framework. This approach exhibits distinctive advantages in addressing intricate boundary conditions and enables direct, precise simulation of internal flow fields within porous structures characterized by complex geometries. Specifically, for CLs possessing nanoscale porosity, where oxygen transport occurs predominantly under low Reynolds number regimes, the mass transfer processes are chiefly governed by diffusive mechanisms. Consequently, the transport behavior of oxygen through these CLs can be effectively described by the following governing equation [38]:

(5) $$\frac{\partial C_{O_{2}}}{\partial t}=\nabla •\left(D_{O_{2}}\nabla C_{O_{2}}\right)+S_{O_{2}}$$

where *C_O_*_2_ is the oxygen concentration, *t* is time, *D_O_*_2_ is the oxygen diffusion coefficient, and *S_O_*_2_ is the source term accounting for oxygen consumption due to the electrochemical reaction.

Within this investigation, the D3Q7 LB model was employed to simulate the mass transfer processes of oxygen within the CL. This model encapsulates the transport phenomena through a set of seven discrete velocity vectors, thereby characterizing the anisotropic diffusion behavior inherent in porous media. The temporal evolution of the oxygen concentration field is governed by the following evolution equation:

(6) $$g_{i}(x+c_{i}\Delta t,t+\Delta t)=g_{i}(x,t)-\omega [g_{i}(x,t)-g_{i}^{eq}(x,t)]+w_{i}S_{O_{2}}$$

In this context, *ω* represents the relaxation frequency, while *c_i_* signifies the discrete lattice velocity vectors. The weight coefficient *w_i_* corresponds to each velocity direction, and the equilibrium distribution function *g^eq^* is defined as follows:

(7) $$g_{i}^{eq}=\frac{C}{7},i=0-6$$

(8) $$c_{i}=\left\{\begin{array}{l} (0,0,0),i=0\\ (\pm 1,0,0),(0,\pm 1,0),(0,0,\pm 1),i=1-6 \end{array}\right.$$

The calculation method of oxygen concentration (*C*) is as follows

(9) $$C=\sum_{i=0}^{6}g_{i}$$

S*_O_*_2_, functioning as a sink term for oxygen consumption, necessitates the differentiation between active sites and inactive sites due to pronounced disparities in reaction kinetics and local pore morphology:

(10) $$S_{O_{2}}=\left\{\begin{array}{lll} -\frac{Hni}{RT\Delta L•4F}, & \mathrm{Active}\;\mathrm{site} & \\ 0, & \mathrm{Inactive}\;\mathrm{site} & \end{array}\right.$$

Within the electrochemical reaction system, several pivotal physical parameters are intertwined: *R* denotes the universal gas constant, *T* signifies the absolute temperature, Δ*L* represents the lattice discretization resolution, *F* stands for Faraday’s constant, and *n* enumerates the adjacent active Pt sites. The population of these active sites critically influences the interfacial contact area and, consequently, the catalytic efficacy of the reaction. The electrochemical reaction rate, *i*, can thus be rigorously quantified through the following expression:

(11) $$i=\frac{i_{ref}C_{O_{2},i}}{C_{O_{2},ref}}\exp\left(\frac{\alpha F\eta }{RT}\right)$$

Herein, *i_ref_* denotes the reference current density, while *C_O_*_2,*i*_ and *C_O_*_2,*ref*_ represent the local oxygen concentration within the CL and the reference oxygen concentration, respectively. During electrochemical reactions, oxygen molecules initially dissolve into ionomer phase, followed by diffusion towards the Pt particle surfaces where they participate in proton-coupled reduction reactions. Notably, due to the substantial discrepancy in oxygen diffusivity between the CL porous structure and the ionomer matrix, a conspicuous step change in oxygen concentration emerges across the ionomer/pore interface. This interfacial oxygen concentration gradient can be quantitatively described by the following relationship:

(12) $$\frac{RT}{H}=\frac{C_{O_{2},i}}{C_{O_{2},pore}}$$

*C_O_*_2,*pore*_ is the oxygen concentration in the pores, and *H* is the Henry constant, which is only related to temperature:

(13) $$H=0.101325e^{\left(\frac{-666}{T}+14.1\right)}$$

Moreover, considering the disparity in oxygen diffusivity between the CL pores and the ionomer phase, a critical step in modeling oxygen transport at varying spatial locations involves the precise determination of the effective diffusion coefficient. Within the porous domain, the combined influences of bulk diffusion and Knudsen diffusion necessitate concurrent consideration due to the characteristic pore dimensions. The respective formulations governing these dual transport mechanisms are articulated as follows:

(14) $$D_{\mathrm{bulk}}=\frac{2.2\times 10^{-5}{\left(\frac{T}{293.2}\right)}^{1.5}}{\left(\frac{P}{1.013\times 10^{5}}\right)}$$

(15) $$D_{Kn}=\frac{d_{p}}{3}{\left(\frac{8RT}{\pi M_{O_{2}}}\right)}^{0.5}$$

(16) $$D_{pore}={\left(\frac{1}{D_{bulk}}+\frac{1}{D_{Kn}}\right)}^{-1}$$

Integral to this calculation, *d_p_* signifies the effective pore diameter, ascertained through an iterative expansion procedure along the pore lattice vectors until a non-pore node is encountered. Subsequently, the mean value across all explored directions is computed to establish the representative pore diameter. The oxygen diffusivity within the ionic polymer matrix can be derived using the following expression [39]:

(17) $$D_{i}=10^{-10}(0.1543(T-293)-1.65)$$

When the diffusion coefficients of oxygen in pores and ionomers are obtained, the relaxation frequency *ω* in Equation (6) can be calculated:

(18) $$\omega ={\left(3D+0.5\right)}^{-1}$$

### 2.3. Computational Domain and Boundary Conditions

This study adopts a computational domain of 150 × 150 × 1069 lattice units (lu^3^), which corresponds to a physical volume of 450 × 450 × 3207 nm^3^, thereby ensuring an accurate representation of the CL’s intricate mesostructure. To simulate realistic surface interactions, a half-way bounce back boundary condition is implemented at the surfaces of carbon supports and Pt nanoparticles, effectively replicating diffusive reflections. Following standard practices for periodic structure modeling, the four boundaries parallel to the z-axis are designated as periodic, enabling the continuous propagation of transport phenomena across these planes. Notably, the initial lattice plane along the *z*-direction (*z* = 1 lu) is configured as the gas inlet boundary, analogous to the interface between the GDL and the CL. At the inlet, the oxygen concentration is maintained constant at *C_O_*_2,*in*_, establishing a Dirichlet boundary condition that drives the mass transfer process. From this foundational setup, the unknown distribution function *g*_6_ at the inlet can be systematically deduced through the following methodology:

(19) $$g_{6}=(w_{5}+w_{6})C_{O_{2},in}-g_{5}$$

The last-layer lattice in the *z* direction, that is, the *z* = 1069 lu plane, is set as the gas outlet. This plane is regarded as the cross-section of CL. By applying the open boundary condition, the unknown distribution function *g*_5_ can be calculated.

(20) $$g_{5}(x,y,1069)=g_{5}(x,y,1068)$$

To accurately simulate the concentration jump effect of oxygen at the interface between ionomers and pores, the boundary conditions of the ionomer/pore interface region need to be set as follows:

(21) $$g_{i}(x_{p},t+\Delta t)=\frac{1}{-\frac{1}{4k_{dis}}\frac{\Delta x}{\Delta t}-\frac{1}{H}-1}\left[\frac{1}{-\frac{1}{4k_{dis}}\frac{\Delta x}{\Delta t}+\frac{1}{H}-1}{\hat{g}}_{i}(x_{p},t+\Delta t)-2{\hat{g}}_{i}(x_{i},t+\Delta t)\right]$$

(22) $$g_{i}(x_{p},t+\Delta t)=g_{i}(x_{i},t+\Delta t)=\frac{1}{-\frac{1}{4k_{dis}}\frac{\Delta x}{\Delta t}-\frac{1}{H}-1}\left[\frac{1}{-\frac{1}{4k_{dis}}\frac{\Delta x}{\Delta t}-\frac{1}{H}+1}{\hat{g}}_{i}(x_{i},t+\Delta t)-\frac{2}{H}{\hat{g}}_{i}(x_{p},t+\Delta t)\right]$$

Within this context, *x_p_* and *x_i_* respectively denote the pore nodes and ionomer nodes situated at the interface between the ionomer phase and the porous domain, while *g_i_* signifies the post-collision distribution function, $\bar{i}$, with *i* representing the counterpart associated with the opposing velocity direction.

In advancing the LB modeling framework, a pivotal step involves the determination of conversion coefficients to bridge the discretized lattice units with physical quantities. The length conversion coefficient, embodied by the lattice resolution Δ*_l_* = 3 × 10^−9^ m, establishes the spatial scaling. The concentration conversion factor, Δ*_C_*, is judiciously selected as 1 mol/m^3^ to align with the simulation’s requirements. To uphold numerical stability, the minimal oxygen diffusivity within the ionomer matrix is leveraged to compute the diffusion coefficient conversion factor, yielding Δ*_D_* = 5.7413 × 10^−8^ m^2^/s. This value ensures that the diffusive processes remain within the model’s stability bounds. Subsequently, the temporal conversion coefficient, Δ*_t_*, is derived from Δ*_l_* and Δ*_D_*, resulting in Δ*_t_* = 1.5676 × 10^−10^ s, harmonizing the time scales of the lattice with physical reality. Finally, the source term conversion coefficient, Δ*_S_*, is deduced from Δ*_C_* and Δ*_t_*, quantified as Δ*_S_* = 6.3793 × 10^9^ mol/(m^3^·s), thereby encapsulating the transformation of reaction kinetics into the lattice-based framework.

## 3. Results and Discussion

In this study, three distinct gradient distribution patterns of carbon support volume fraction within the CL were systematically investigated, including LG, SG, and HSG configurations. These were benchmarked against a uniform CL. Furthermore, two directional trends in the local carbon volume fraction along the through-plane direction were considered: increasing (positive gradient, P) and decreasing (negative gradient, N), along with two gradient magnitudes: small (S) and large (L). For a given distribution form and gradient magnitude, the structures with increasing and decreasing carbon volume fractions are geometrically symmetric about the mid-plane of the CL.

Beyond material-level innovations such as quantum dots and MXenes [40], the structural engineering of conventional catalyst layers offers an alternative and complementary pathway for performance enhancement. Before presenting the gradient-structure results, we briefly address the validation of the present LBM model. The model has been rigorously validated in our previous work [38], where the predicted oxygen concentration in the ionomer phase was compared with the benchmark data of Hou et al. [34] under identical CL compositions. Good agreement was obtained, confirming that the model reliably captures the coupled oxygen diffusion and electrochemical reaction processes. For brevity, those validation details are not reiterated here; details can be found in our earlier work [38].

In the following discussion, an abbreviation scheme is adopted to denote each configuration concisely, such as LG/SG/HSG-N/P-S/L, where LG/SG/HSG indicate the distribution type, N/P represent the direction of the carbon volume fraction gradient, and S/L refer to the gradient magnitude. A total of 13 CL structures with different gradient configurations were considered, and their structural characteristics are summarized in Table 1. Here, *ε_C-max_* and *ε_C-min_* denote the maximum and minimum local carbon volume fractions, respectively. All gradient structures exhibit comparable overall volume fractions of carbon, platinum, and ionomer, as well as similar total thickness. Relative to the baseline case, the deviation in total porosity is within 0.0058, the maximum relative error in ECSA is only 0.41%, and the pore connectivity remains above 99.6% for all cases [17]. These comparable structural parameters effectively minimize the influence of randomness introduced by the stochastic reconstruction algorithm, thereby allowing a reasonable attribution of performance variations primarily to the anisotropic features of the through-plane porosity gradient.

In Figure 2, the pore size distribution characteristics of gradient CLs are presented. Figure 2a–c show that all gradient configurations exhibit unimodal pore size distributions, with peak pore diameters in the range of 20–40 nm, consistent with the mesoporous nature of typical CLs [41]. Compared to the uniform structure, gradient designs significantly alter the peak frequency and distribution width, indicating that gradient configurations affect the statistical pore structure by modulating the local packing density of carbon supports. For larger gradient magnitudes, the peak frequency is reduced and the peak position shifts toward larger pore diameters, suggesting that increased gradient strength broadens the distribution and intensifies local aggregation, leading to the formation of more macropores. In the case of SG, the presence of shoulder peaks reflects abrupt pore transitions at layer interfaces, consistent with phase discontinuities across segmented domains. Notably, high-gradient SG and HSG structures exhibit a pronounced increase in large pore fractions, revealing that gradient type modulates the magnitude effect.

Figure 2d–f further show that gradient designs substantially reshape the spatial distribution of pore sizes. In LG, negative gradients lead to a reduction in average pore size near the membrane side to approximately 12 nm, forming a continuous tapering profile; large gradients extend the pore size range to 13 nm. In SG structures, a significant jump in pore size occurs near the 1600 nm interface; for example, the SG-N-L case shows a sharp pore size shift of up to 64%, indicating enhanced structural reorganization at the discontinuous interface. HSG structures exhibit a three-segment response: in positive gradients, the membrane-side constant region maintains small pores, while the intermediate linear segment exhibits positive or negative slopes depending on the direction. In HSG-P-L, the pore size increases by as much as 42% at the GDL interface. It is noteworthy that positive large-gradient structures maximize the pore size near the GDL side across all configurations. These results indicate that the gradient direction effectively controls the mass transport pathways by modulating carbon support density, where larger pores near the GDL side enhance gas diffusion capabilities.

Figure 3 systematically analyzes the influence of carbon support gradient distributions on the spatial characteristics of the local ECSA within the CL, demonstrating that gradient design significantly modulates the spatial distribution of ECSA. As shown in Figure 3a, the ECSA of LG structures exhibits a continuous variation along the through-plane direction. In positive gradients, the ECSA near the membrane side increases by nearly 40% compared to the GDL side, whereas negative gradients show the opposite trend. In Figure 3b, SGs induce abrupt interfacial transitions. For instance, SG-P-L exhibits an ECSA fluctuation exceeding 12,000 nm^2^ across the thickness, and negative gradients display higher ECSA near the GDL side relative to the membrane side. Figure 3c shows that HSGs enable coordinated control via a three-segment structure. In HSG-P-L, the ECSA remains at approximately 22,000 nm^2^ near the GDL side and increases to over 30,000 nm^2^ toward the membrane side.

The gradient direction dictates the location of the high-activity region: positive gradients enhance catalytic activity near the membrane, while negative gradients favor electrochemical reactions near the GDL. The gradient magnitude influences the extent of ECSA variation; larger gradients lead to greater fluctuations, with ECSA standard deviations up to three times those of the uniform structure (e.g., LG-P-L), but also yield higher peak activity. In contrast, small gradients exhibit narrower ECSA variation, offering improved spatial stability. Collectively, the distribution form, gradient direction, and gradient magnitude are key determinants of ECSA morphology. These findings confirm that precisely engineered carbon volume fraction gradients can achieve spatially targeted optimization of catalytic activity, offering a theoretical foundation for the rational design of high-performance electrode architectures.

Figure 4 presents the steady-state oxygen concentration distributions obtained from LB simulations of oxygen transport and consumption at active sites, illustrating the directional regulation of mass transport pathways induced by carbon support gradient distributions. In positive gradient configurations, regions of high *ε_C_* are concentrated near the membrane side (right side), where low-oxygen-concentration zones (blue regions) are markedly reduced. As the gradient magnitude increases to level L, these low-oxygen zones become fragmented into discrete patches, while a broad high-concentration region is maintained near the GDL side. This confirms that positive gradient designs, by increasing carbon support density at the membrane side, compress the nanoporous network, thereby enhancing Knudsen diffusion efficiency and overcoming conventional oxygen transport limitations. In contrast, negative gradient configurations display complementary characteristics, with high-*ε_C_* zones located near the GDL side. In SG-N-L, the formation of a dense carbon agglomerate network near the GDL results in an abrupt drop in local oxygen concentration at the interface where *ε_C_* changes sharply. The impact of gradient type is also evident; the SG induces concentration discontinuities at the interfacial transition, whereas the HSG enables smooth concentration gradients via a linear transition zone between 1000–2000 nm, thereby mitigating the risk of localized transport bottlenecks. HSG-P configurations achieve dual-region optimization; the GDL side high-oxygen region ensures robust gas supply, while the fraction of low-oxygen-concentration regions near the membrane side is reduced to 22%, a 40% decrease compared to the uniform structure. This performance enhancement is attributed to the dual reconstruction mechanism of the pore network induced by the support gradient. High-*ε_C_* zones generate finer nanopores that improve diffusion efficiency, while low-*ε_C_* regions feature more open, connected pore channels that reduce flow resistance.

To quantitatively characterize the oxygen transport behavior, the through-plane oxygen concentration profiles at steady state are presented in Figure 5. Overall, oxygen concentration decreases along the thickness direction in all cases; however, the distribution form, gradient direction, and gradient magnitude exert significant regulatory effects on the transport process. As shown in Figure 5a, for LG structures, LG-P-L maintains a relatively high oxygen concentration of approximately 0.85 mol/m^3^ near the membrane side, while LG-N-L exhibits a much lower value of around 0.40 mol/m^3^, indicating that positive gradients markedly enhance oxygen availability at the membrane interface. In stepwise gradient structures, SG-P-L also outperforms SG-N-L, with terminal concentrations of approximately 0.65 mol/m^3^ and 0.28 mol/m^3^, respectively, suggesting that oxygen concentration responses beyond the structural transition are closely related to the gradient configuration. HSG demonstrates superior performance through spatially coordinated regulation. For instance, HSG-P-L maintains a nearly uniform concentration distribution along the thickness, with an oxygen concentration of approximately 0.95 mol/m^3^ at z = 2000 nm, whereas HSG-N-L drops to around 0.45 mol/m^3^ at the same location. This further underscores the advantage of positive gradients in strengthening oxygen supply from the GDL side and reducing overall mass transport resistance. From the perspective of gradient direction, positive gradients enhance the porosity near the GDL side, facilitating oxygen ingress and sustaining higher concentrations along the reaction front. In contrast, negative gradients introduce substantial transport resistance at the inlet, leading to rapid concentration decay in the front region and resulting in a steep oxygen concentration gradient that impairs oxygen delivery to the membrane side. In terms of gradient magnitude, large gradients offer greater potential for regulating transport pathways. For example, HSG-P-L exhibits a lower concentration gradient across the CL compared to HSG-P-S. At z = 3000 nm, the oxygen concentration in HSG-P-S is approximately 0.62 mol/m^3^, whereas HSG-P-L maintains a higher value above 0.75 mol/m^3^. This verifies the synergistic effect between gradient strength and distribution form in enhancing oxygen transport performance.

Figure 6a–c illustrate the through-plane distributions of oxygen consumption rate within the CL under different carbon support gradient strategies. Across all configurations, the oxygen consumption rate generally decreases from the GDL side to the membrane side, driven by the coupled effects of oxygen concentration, ECSA, and local pore structure. At any given thickness, differences in the consumption rate reflect the degree of local reaction activation and the efficiency of oxygen transport, both of which are dictated by the structural configuration. In Figure 6a, the LG-P-L structure exhibits the most gradual decline in oxygen consumption rate, maintaining values above 1.0 × 10^−8^ mol/(m^3^·s) at z = 2000 nm, significantly outperforming both the uniform structure (~0.85 × 10^−8^ mol/(m^3^·s)) and LG-N-L (<0.6 × 10^−8^ mol/(m^3^·s)). This advantage can be attributed to the sustained high oxygen concentration throughout the CL in LG-P-L, particularly near the membrane side where the concentration remains above 0.85 mol/m^3^, in Figure 5a. In parallel, Figure 3a shows a slight increase in ECSA toward the membrane, jointly supporting a high level of reaction activity in that region. In contrast, LG-N-L has a higher carbon volume fraction and reduced porosity near the GDL side, which impedes initial oxygen diffusion. Although a transient peak in reaction rate is observed near the inlet, it quickly declines due to rapid oxygen depletion and reduced ECSA. Figure 6b highlights the regulatory effect of abrupt structural transitions in stepwise gradients. SG-P-L exhibits a pronounced peak in oxygen consumption rate (~1.35 × 10^−8^ mol/(m^3^·s)) at the transition interface, driven by a local increase in carbon volume fraction that enlarges the pore size and enhances channel accessibility. This peak is supported by the local oxygen concentration spike in Figure 5b and the corresponding rise in ECSA in Figure 3b, collectively enhancing the localized reaction rate. By contrast, SG-N-L shows a steep decline in consumption rate beyond the interface due to elevated carbon content and contracted pore channels in the downstream region, leading to oxygen concentrations below 0.5 mol/m^3^. Despite relatively high ECSA in the upstream section, the sharp drop in downstream ECSA limits overall reaction depth. In Figure 6c, the HSG configuration achieves a synergistic match between oxygen transport and reaction activity through multi-zone structural design. HSG-P-L exhibits the most uniform oxygen consumption profile across the CL, with values above 1.0 × 10^−8^ mol/(m^3^·s) maintained even at z = 2500 nm. As shown in Figure 5c, this structure sustains oxygen concentrations as high as 1.5 mol/m^3^ in the first segment and above 1.2 mol/m^3^ in the middle segment, values considerably higher than those in the uniform or negative-gradient cases. Meanwhile, Figure 3c shows minimal ECSA variation across zones, consistently ranging between 25,000 and 27,000 nm^2^, demonstrating strong structure–function integration. This gradient configuration ensures sufficient oxygen supply in the front region while avoiding oxygen-depleted zones downstream, thereby improving the overall utilization efficiency of the reactive region.

The above observations reveal a consistent mechanistic picture linking gradient direction to electrochemical performance. In positive gradient configurations, the higher carbon volume fraction near the membrane side leads to two concurrent effects. First, it increases the local density of catalytic sites, as reflected by the elevated ECSA in that region (Figure 3). Second, the accompanying pore structure modulation, with finer nanopores near the membrane and more open pores near the GDL side (Figure 2), facilitates oxygen supply from the GDL while maintaining high reactant availability at the reaction front. This combination results in a more uniform oxygen concentration distribution (Figure 5) and sustains the oxygen consumption rate throughout the CL thickness (Figure 6). In contrast, negative gradients concentrate carbon near the GDL side, which, while enhancing local ECSA near the inlet, creates a dense structure that impedes oxygen penetration and leads to rapid oxygen depletion downstream, thereby limiting the reaction depth and overall current output. The uniform CL, serving as a reference case with no gradient (i.e., equilibrium distribution without directional bias), exhibits intermediate performance between the positive and negative gradient extremes. This comparison confirms that introducing a favorable gradient direction is essential for breaking the inherent trade-off between oxygen transport and catalytic activity in conventional homogeneous CLs.

Figure 7 presents the current densities corresponding to various carbon support volume fraction distribution strategies. The uniform CL exhibits a current density of approximately 611.88 mA/cm^2^. All positive gradient configurations outperform this baseline, with LG-P-L and HSG-P-L achieving the highest values of 692.56 mA/cm^2^ and 694.11 mA/cm^2^, respectively. In contrast, all negative gradient configurations yield lower current densities than the uniform structure, with HSG-N-L performing the worst at around 400 mA/cm^2^. This trend can be attributed to the structural advantage of positive gradients, in which a lower carbon volume fraction is assigned near the GDL side. This design increases local porosity, facilitates oxygen transport, and when combined with higher oxygen concentrations and improved ECSA, substantially enhances reaction intensity. Conversely, negative gradients suffer from hindered transport near the GDL side and oxygen depletion in the downstream region. Even with localized ECSA enhancement, these configurations fail to sustain effective deep-layer electrochemical reactions.

To clarify the dominant factor in performance improvement, we compare the variation ranges of ECSA and oxygen concentration across all configurations. As shown in Table 1, the overall ECSA varies by less than 0.41%, whereas the local oxygen concentration near the membrane varies by more than threefold (Figure 5). This suggests that the performance gain is primarily driven by enhanced oxygen transport rather than by an increase in total catalytic sites.

Regarding gradient magnitude, large gradients consistently outperform small ones. HSG-P-L achieves optimal coupling of oxygen concentration, ECSA, and reaction rate through its three-segment design, yielding the highest current output. However, its current density advantage over LG-P-L is marginal (~1.55 mA cm^−2^), and this modest gain alone may not justify the added structural complexity. HSG-P-L does offer additional benefits, more uniform oxygen distribution and sustained reaction rate across the CL (Figure 5c and Figure 6c), which may favor long-term durability. The choice between HSG and LG thus depends on the trade-off between fabrication simplicity and performance uniformity. Among negative gradients, HSG-N-L performs worst because its intermediate transition zone prolongs the low-oxygen region after the initial GDL-side transport barrier, leaving even less oxygen for the membrane-side low-carbon region compared to LG-N-L or SG-N-L (Figure 5c).

Negative gradients underperform even the uniform CL because the high carbon fraction near the GDL creates a dense structure that impedes oxygen ingress (Figure 5). Although ECSA is high near the inlet (Figure 3), rapid oxygen depletion leaves insufficient reactant for the membrane-side reaction zone, limiting overall reaction depth.

Graded CLs have been successfully fabricated via layer-by-layer deposition [17] and continuous coating [18]. HSG can be realized through three sub-layers (high carbon near membrane, intermediate transition, low carbon near GDL), while LG is achievable by continuously varying slurry composition [18]. Precise nanoscale control remains challenging, but the performance benefits identified here motivate further experimental development.

We acknowledge that proton transport, liquid water, and ionomer distribution also affect CL performance, especially at high current densities. With a fixed ionomer-to-carbon ratio, proton conductivity is not explicitly optimized here, though larger pores near the GDL in positive gradients may facilitate water drainage. These coupled effects warrant future investigation.

Regarding durability, the more uniform oxygen distribution in positive gradients (Figure 5) may mitigate localized starvation and associated carbon corrosion [9,10]. The spatial matching of ECSA with oxygen availability could reduce local overpotential gradients that drive Pt degradation [25], while broader pores near the GDL may aid water removal and mitigate flooding. Direct experimental validation under accelerated stress tests remains a future direction.

## 4. Conclusions

This study investigated the effect of through-plane carbon support volume fraction gradients on oxygen transport and electrochemical performance in PEMFC catalyst layers using a pore-scale lattice Boltzmann model. Three gradient configurations, linear (LG), stepwise (SG), and hybrid stepwise (HSG), were examined under varying gradient directions and magnitudes.

The results demonstrate that positive gradients, with increasing carbon fraction toward the membrane, yield higher oxygen diffusivity and electrochemical activity than negative and uniform structures. Among all configurations, the HSG with a positive large gradient (HSG-P-L) exhibits the highest peak current density of 694.11 mA cm^−2^, which is 13.4% higher than that of the uniform CL. This improvement is associated with a broadened pore size distribution that facilitates oxygen transport and an increased electrochemically active surface area near the membrane.

The effect of gradient magnitude on pore structure is dependent on gradient form and direction. Larger gradients produce broader pore size distributions and more pronounced structural heterogeneities. In SG structures, abrupt transitions at layer interfaces can cause localized discontinuities in oxygen transport, whereas the HSG configuration, with its linear transition zone, maintains more continuous mass transport across the CL thickness.

These findings establish a quantitative link between carbon support gradient design and CL performance. The HSG-P-L configuration provides a reference for the design of catalyst layers with improved transport and reaction characteristics for PEMFC applications.

## Figures and Tables

**Figure 1:**
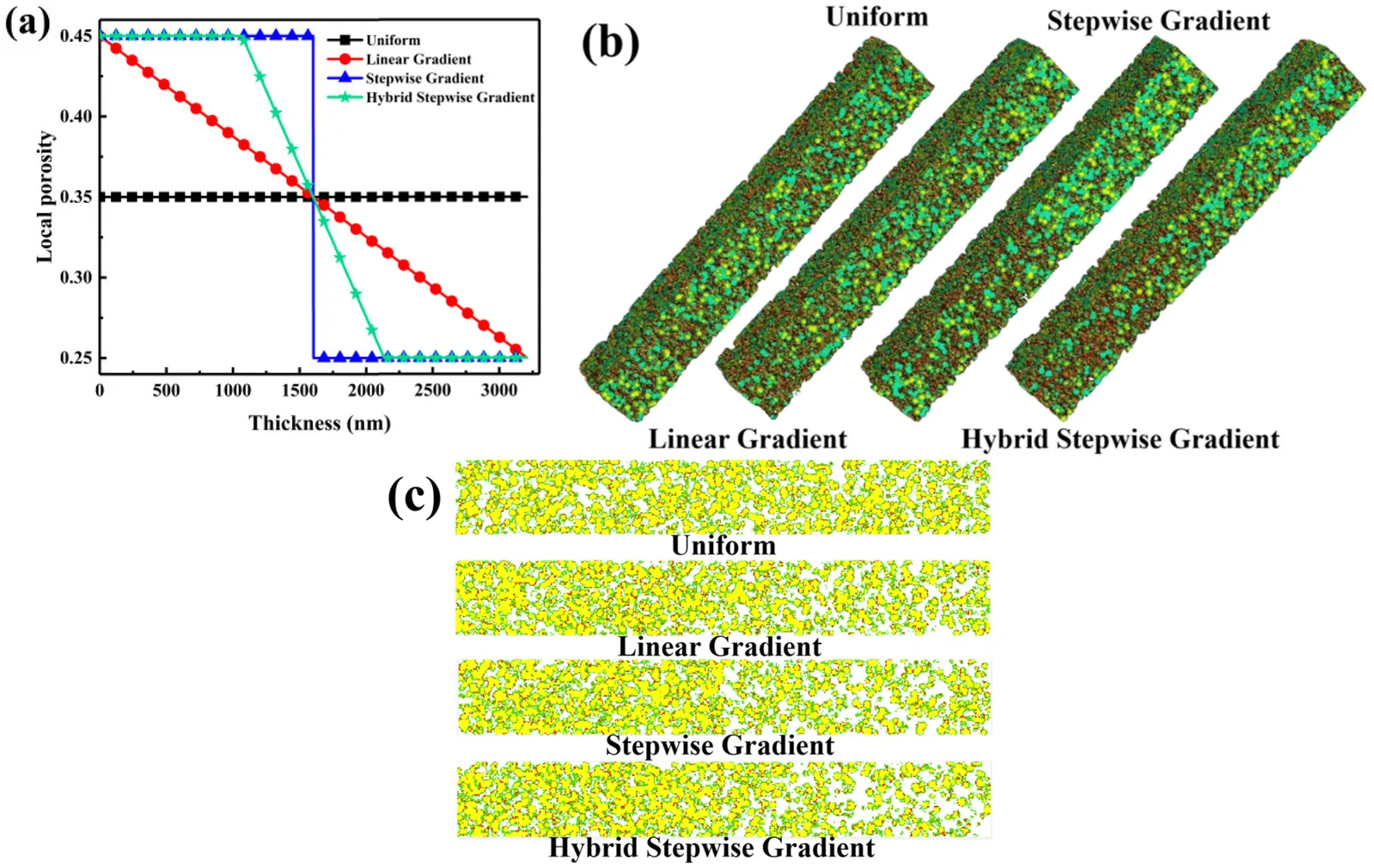
(**a**) Schematic diagram of the distribution of carbon support volume fraction along the thickness direction, and schematic diagrams of CL structures with uniform, linear gradient, stepwise gradient and hybrid stepwise gradient in (**b**) 3D view and (**c**) 2D view.

**Figure 2:**
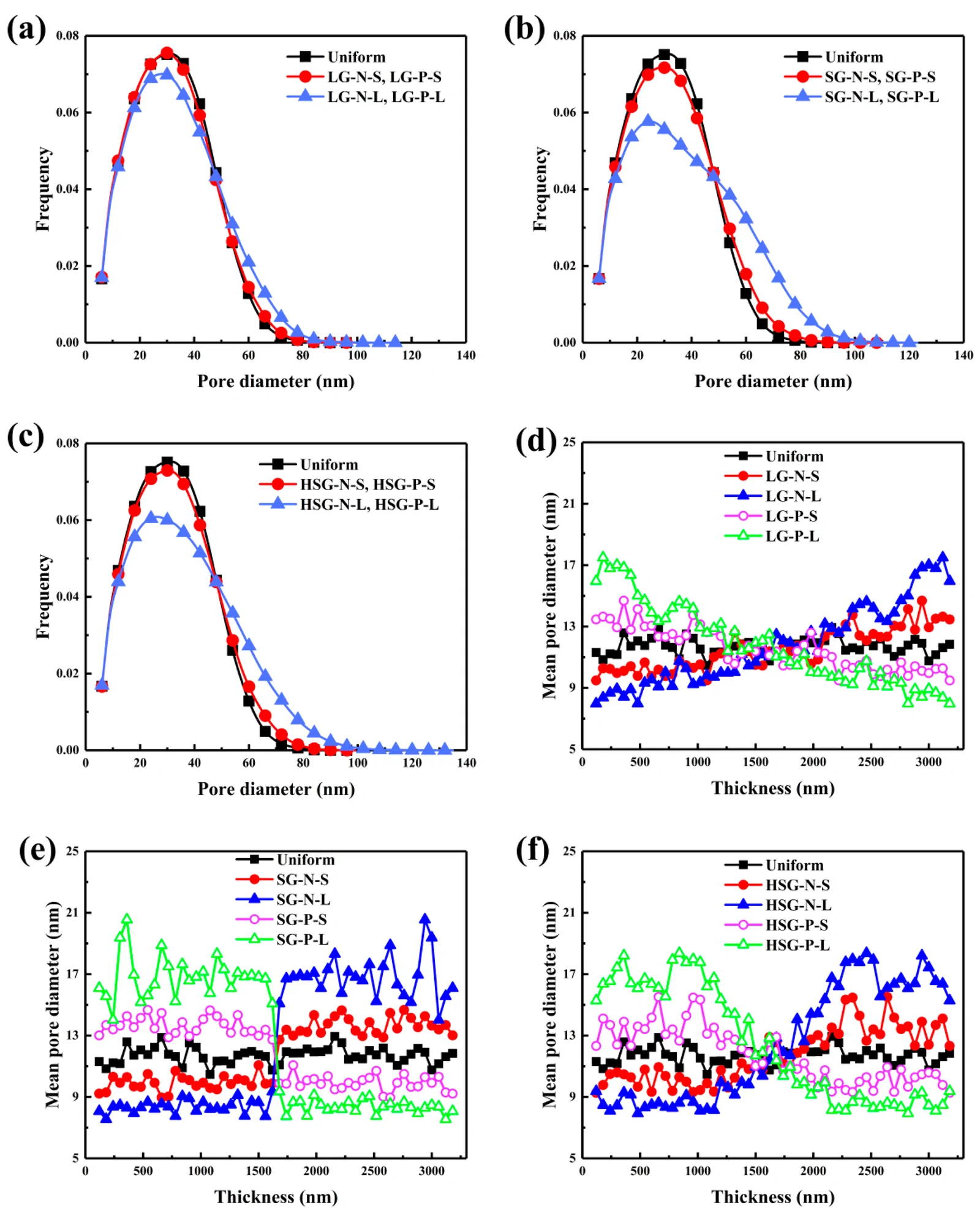
Pore size distribution of gradient CLs. Overall pore size distributions, (**a**) LG, (**b**) SG, (**c**) HSG; local pore size distributions along the through-plane direction: (**d**) LG, (**e**) SG, (**f**) HSG.

**Figure 3:**
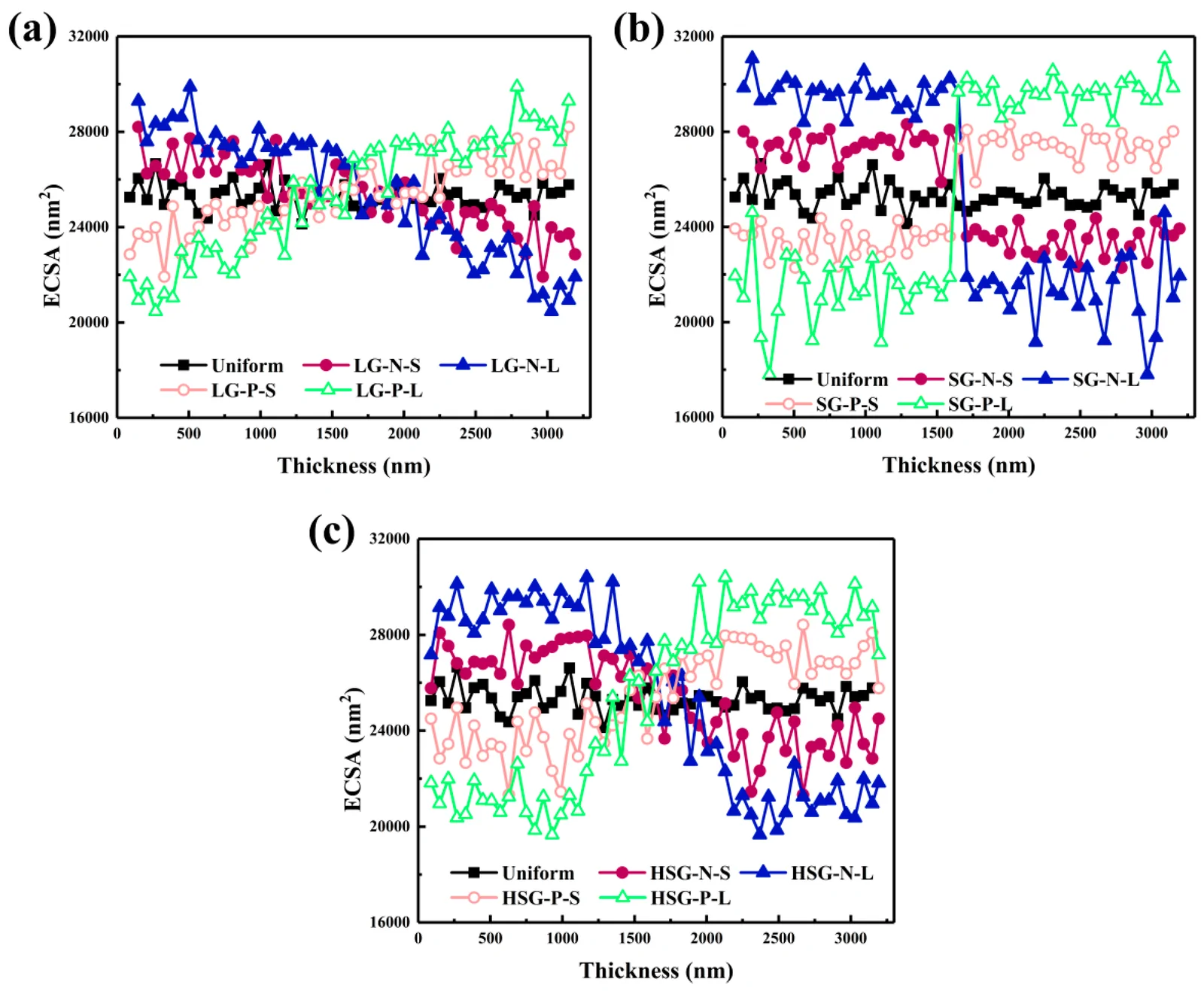
Through-plane distribution of local ECSA in the CL, (**a**) LG, (**b**) SG, (**c**) HSG.

**Figure 4:**
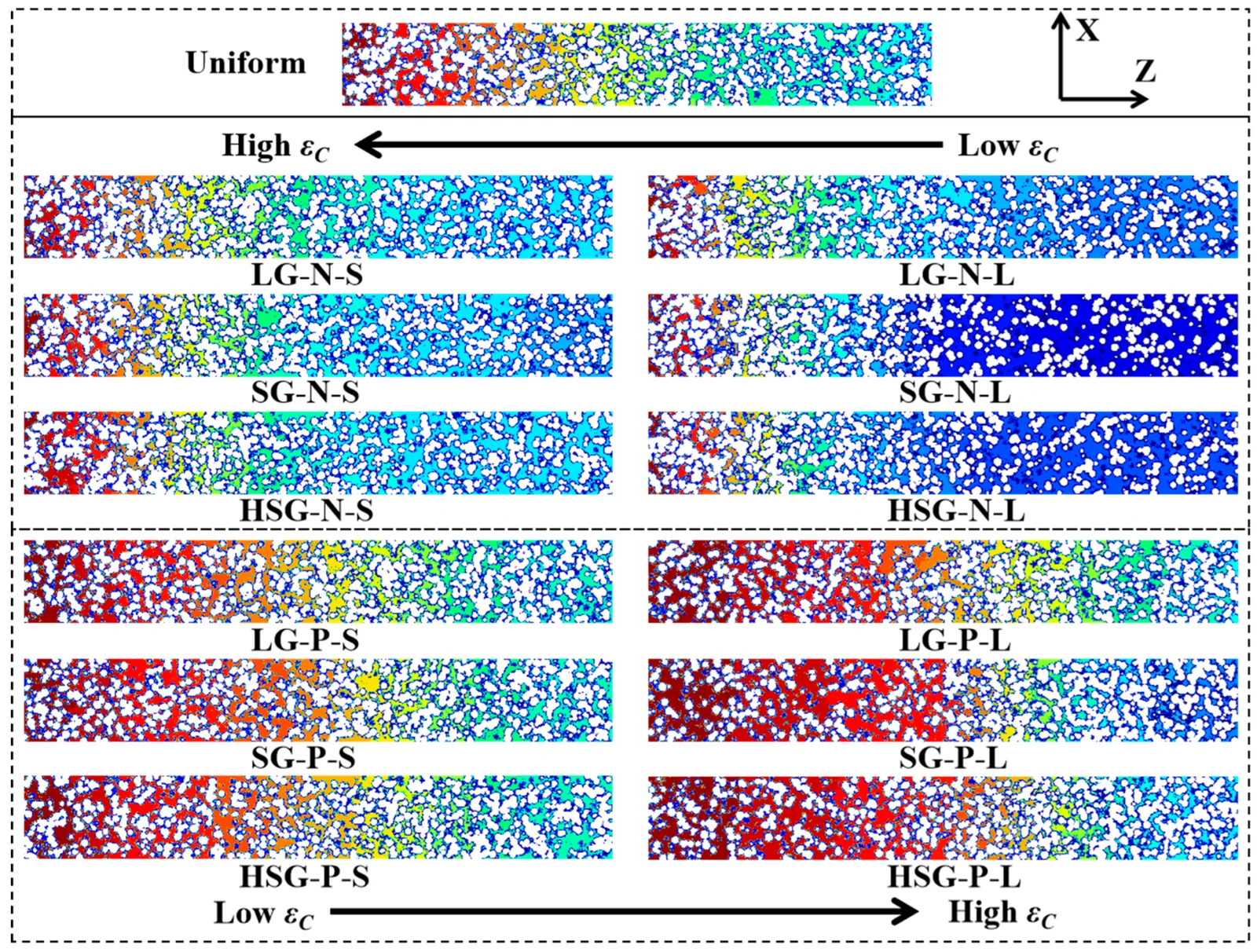
Oxygen concentration contour maps at steady state.

**Figure 5:**
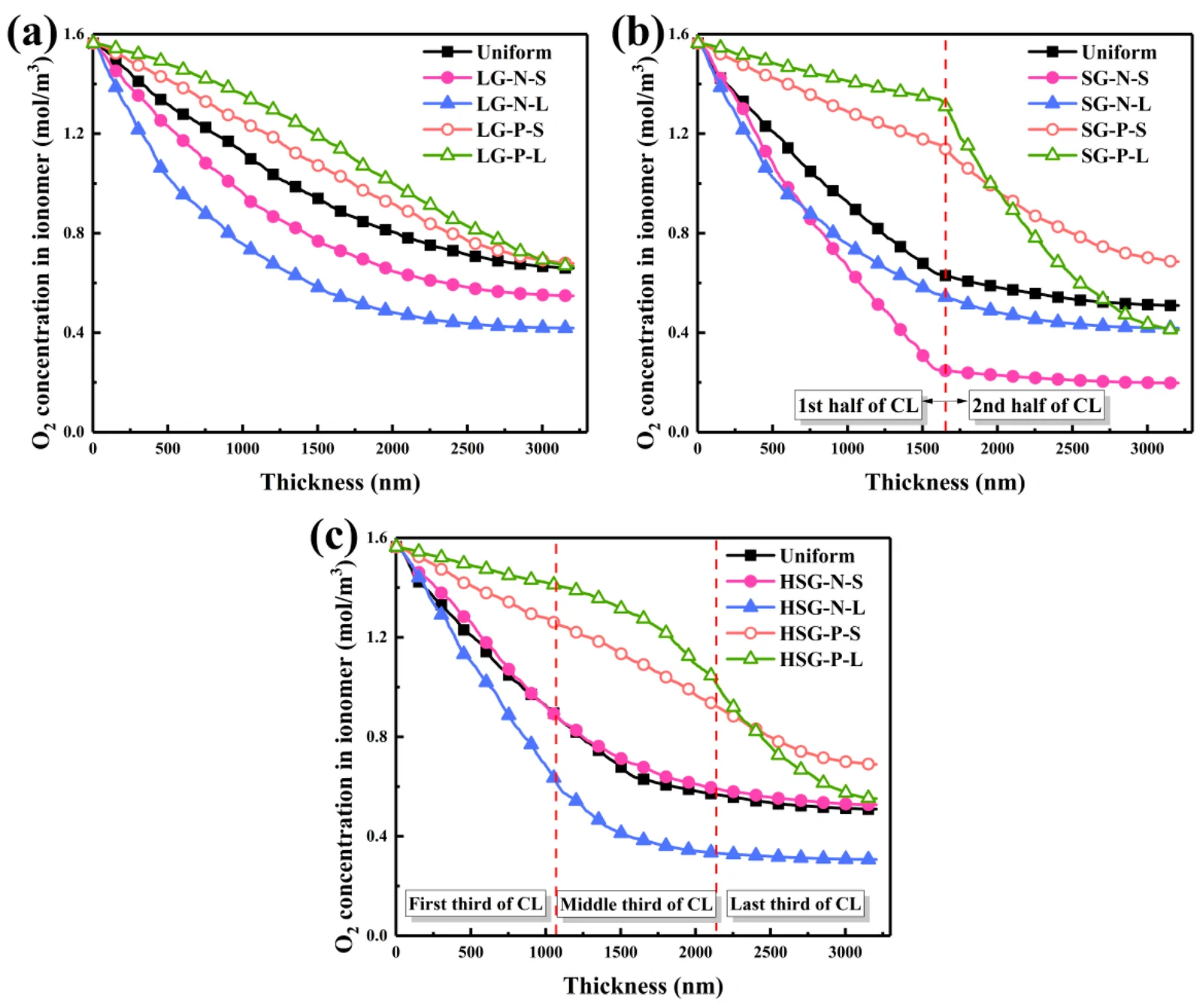
Through-plane distribution of local oxygen concentration within the CL, (**a**) LG, (**b**) SG, (**c**) HSG.

**Figure 6:**
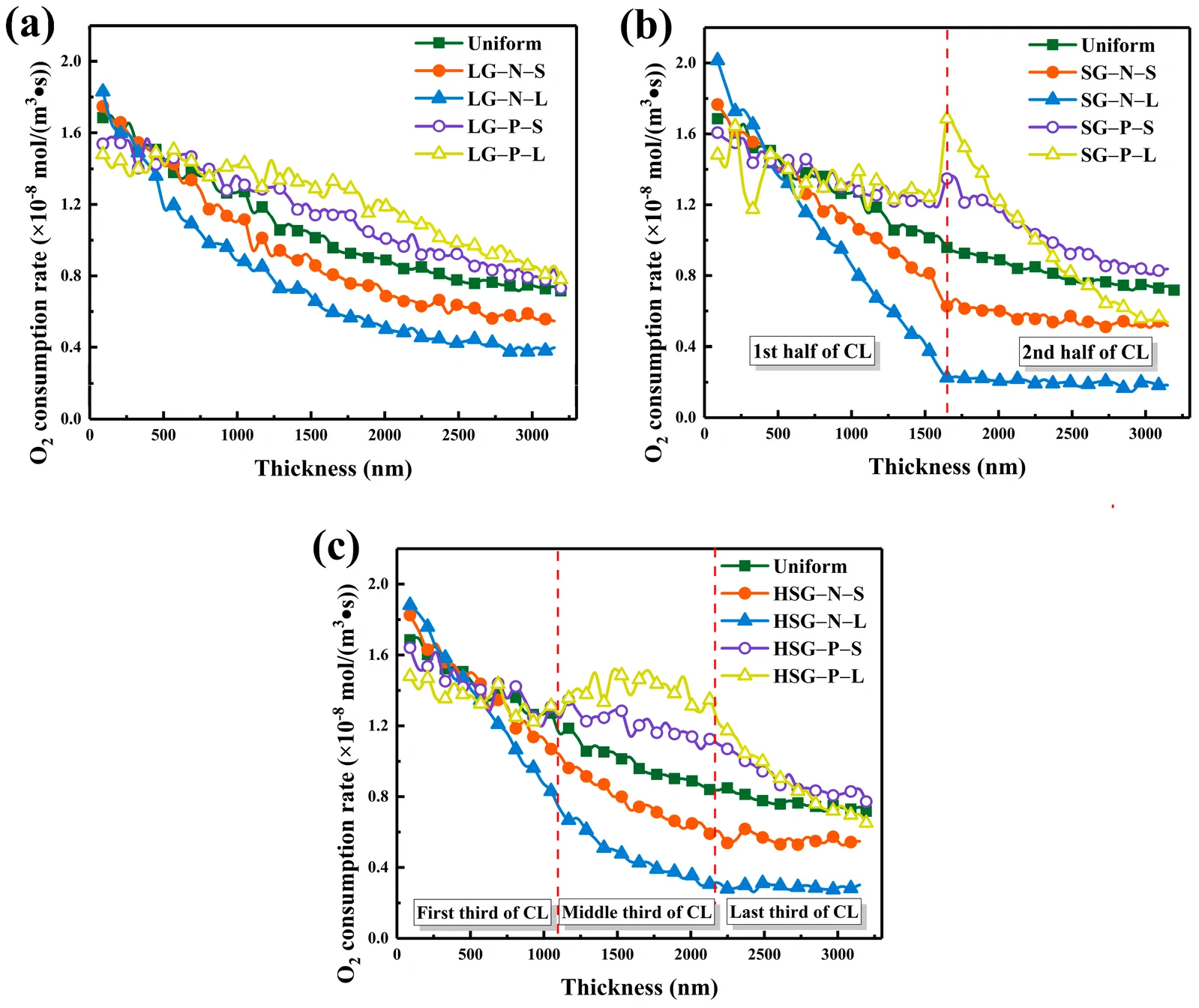
Through-plane distribution of local oxygen consumption rate within the CL, (**a**) LG, (**b**) SG, (**c**) HSG.

**Figure 7:**
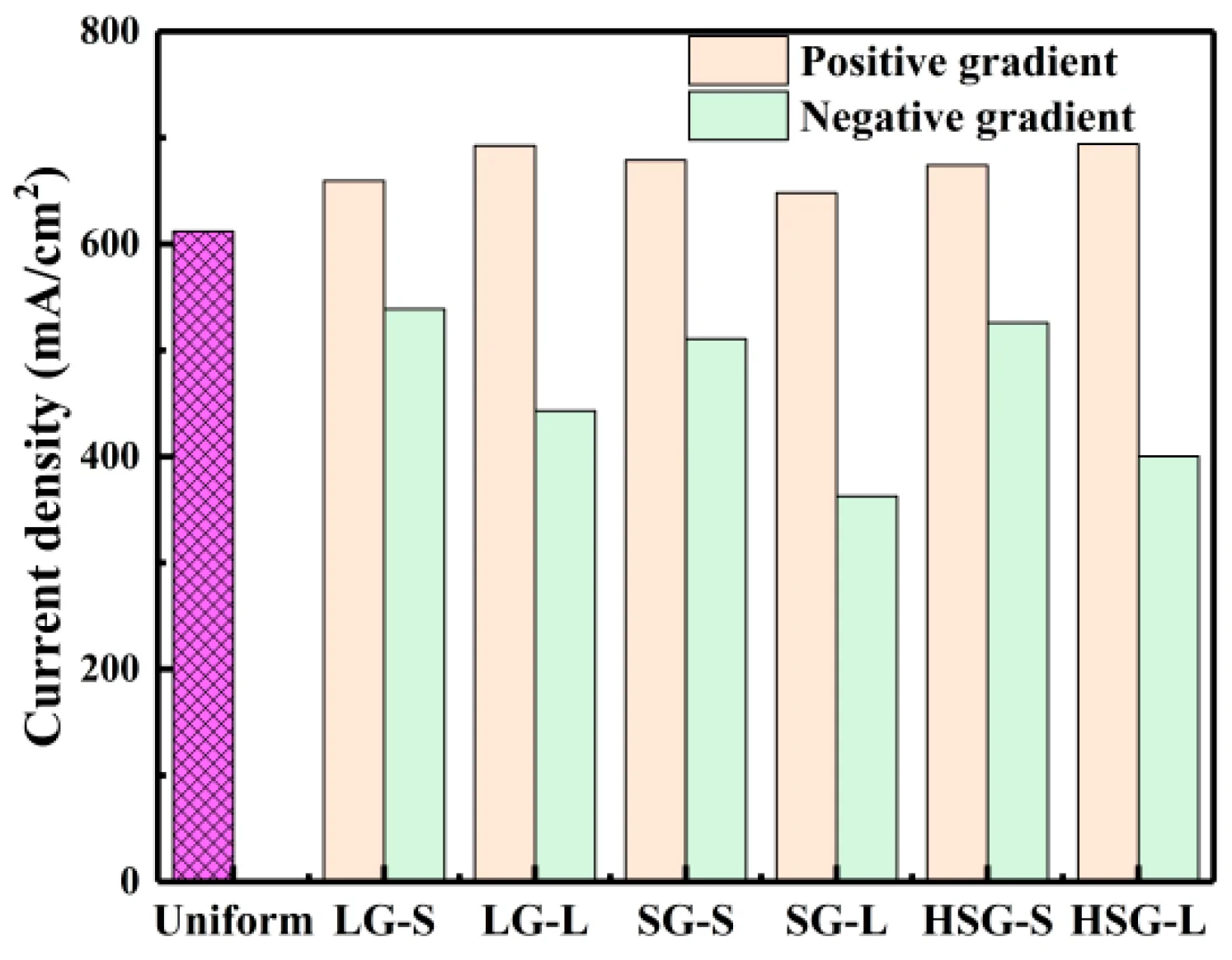
Comparison of current densities for gradient CLs.

**Table 1:** Structural information of uniform and gradient CLs.

Case Number	Carbon Particle Distribution	Porosity	*ε_C_*	*ε_C-max_*	*ε_C-min_*	Thickness (nm)	Connected Rate	ECSA (nm^2^)
1	Uniform	0.3475	0.3465	-	-	3207	99.69%	27,017,991
2,3	LG-N-S, LG-P-S	0.3442	0.3465	0.3965	0.2965	3207	99.66%	27,045,576
4,5	LG-N-L, LG-P-L	0.3469	0.3465	0.4465	0.2465	3207	99.66%	27,074,088
6,7	SG-N-S, SG-P-S	0.3503	0.3465	0.3965	0.2965	3207	99.67%	27,082,152
8,9	SG-N-L, SG-P-L	0.3533	0.3465	0.4465	0.2465	3207	99.63%	27,129,078
10,11	HSG-N-S, HSG-P-S	0.3489	0.3465	0.3965	0.2965	3207	99.67%	27,074,205
12,13	HSG-N-L, HSG-P-L	0.3508	0.3465	0.4465	0.2465	3207	99.63%	27,107,748

## Data Availability

The data presented in this study are available upon request from the corresponding author.
